# Modulating Amyloid-β Toxicity: In Vitro Analysis of Aβ42(G37V) Variant Impact on Aβ42 Aggregation and Cytotoxicity

**DOI:** 10.3390/ijms252313219

**Published:** 2024-12-09

**Authors:** Shu-Hsiang Huang, Shang-Ting Fang, Chin-Hao Yang, Je-Wen Liou, Yi-Cheng Chen

**Affiliations:** 1 Department of Medicine, MacKay Medical College, New Taipei City 252, Taiwan; 2 Department of Biochemistry, School of Medicine, Tzu Chi University, Hualien City 970, Taiwan

**Keywords:** Aβ42, Aβ42(G37V), glycine-zipper motif, aggregation, morphology, toxicity

## Abstract

Alzheimer’s disease (AD) is primarily driven by the formation of toxic amyloid-β (Aβ) aggregates, with Aβ42 being a pivotal contributor to disease pathology. This study investigates a novel agent to mitigate Aβ42-induced toxicity by co-assembling Aβ42 with its G37V variant (Aβ42(G37V)), where Gly at position 37 is substituted with valine. Using a combination of Thioflavin-T (Th-T) fluorescence assays, Western blot analysis, atomic force microscopy (AFM)/transmission electron microscopy (TEM), and biochemical assays, we demonstrated that adding Aβ42(G37V) significantly accelerates Aβ42 aggregation rate and mass while altering the morphology of the resulting aggregates. Consequently, adding Aβ42(G37V) reduces the Aβ42 aggregates-induced cytotoxicity, as evidenced by improved cell viability assays. The possible mechanism for this effect is that adding Aβ42(G37V) reduces the production of reactive oxygen species (ROS) and lipid peroxidation, typically elevated in response to Aβ42, indicating its protective effects against oxidative stress. These findings suggest that Aβ42(G37V) could be a promising candidate for modulating Aβ42 aggregation dynamics and reducing its neurotoxic effects, providing a new avenue for potential therapeutic interventions in AD.

## 1. Introduction

Alzheimer’s disease (AD) is one of the most prevalent neurodegenerative disorders, characterized by a progressive decline in cognitive abilities, such as memory, language, and problem-solving [1,2]. Pathologically, AD is associated with the accumulation of amyloid-β (Aβ) plaques and neurofibrillary tangles composed of hyperphosphorylated tau protein [2]. Aβ peptides, particularly Aβ42, are considered central to AD pathogenesis. According to the amyloid cascade hypothesis, the accumulation of Aβ peptides in the brain initiates a series of events that ultimately lead to neuronal death and cognitive decline [2,3].

Aβ peptides are produced through the proteolytic cleavage of amyloid precursor protein (APP) by β- and γ-secretases [4,5]. Among the Aβ isoforms, Aβ42 is particularly prone to aggregation due to its hydrophobic C-terminal, which promotes the formation of toxic aggregates [6,7,8]. These aggregates are widely believed to disrupt synaptic function and induce apoptosis in neurons through oxidative stress, inflammation, and calcium dysregulation [9,10,11]. Consequently, inhibiting Aβ aggregation or the formation of toxic aggregates has become a primary therapeutic target in AD research [12,13,14]. However, traditional approaches to reducing Aβ42 toxicity, which focus on inhibiting or slowing the aggregation process, have faced significant challenges. Specifically, many therapies fail to differentiate between toxic oligomeric species and the larger, potentially less harmful fibrils. This has led to inconsistent therapeutic outcomes, as inhibiting aggregation may prolong the presence of neurotoxic oligomers rather than eliminating them. Thus, a shift in therapeutic strategy is needed.

One critical mechanism by which Aβ42 exerts its toxicity is generating reactive oxygen species (ROS) and inducing oxidative stress [9,15], a major contributor to the neurodegenerative processes observed in AD. Elevated ROS levels lead to lipid peroxidation [15], DNA oxidation [10], and mitochondrial dysfunction [16], all of which play critical roles in the progression of AD pathology.

Oxidative stress is strongly associated with Aβ42 aggregation. As Aβ42 oligomers interact with neuronal membranes [15,17], they generate ROS, which damages cellular components and triggers a cascade of toxic events. This has led to the development of therapeutic strategies to reduce oxidative stress in AD, alongside efforts to inhibit Aβ42 aggregation [12,13,14]. However, the approaches focused solely on inhibiting aggregation have faced challenges due to the difficulty in targeting specific toxic species, particularly soluble small aggregates.

Two specific regions of the Aβ sequence are particularly critical for its aggregation: the central hydrophobic discordant helix (L17VFFAEDVG25) [18,19,20,21] and the C-terminal glycine-zipper motif (G25, G29, G33, G37) [22,23,24], both of which influence the peptide’s ability to form β-sheet-rich aggregates [18,19,20,21,22,23,24]. Mutations within these regions have been shown to alter Aβ aggregation dynamics and toxicity. For example, replacing L17 and F19 in the discordant helix region with alanine was able to inhibit Aβ40 aggregation and reduce its toxicity [18,19,20,21]. With regards to the glycine-zipper motif, a study by Hung et al. demonstrated that substituting Aβ42 at G25, G29, G33, or G37 with leucine resulted in decreased toxicity in mouse primary cortical neurons compared to wild-type Aβ42 [14]. Further research showed that replacing G37 with leucine could increase the aggregation rate and thus reduce Aβ toxicity in both in vivo and in vitro models [22,23].

A previous study has demonstrated that substituting Gly37 with Val accelerates aggregation, modulates the structural conformation, and changes the morphology of aggregates [24]. Consequently, these modulations lead to the assembly of less toxic aggregates that are less likely to induce cellular damage. These findings suggest that Aβ42(G37V) could represent a novel therapeutic agent, offering a means to modulate Aβ42 aggregation dynamics to reduce its neurotoxic potential.

In this study, we hypothesize that the Aβ42(G37V) variant can alter the aggregation profile of Aβ42, leading to the formation of less toxic aggregates. Using a combination of Thioflavin-T (Th-T) fluorescence assays, Western blotting, atomic force microscopy (AFM), transmission electron microscopy (TEM), and cytotoxicity assays, we evaluate the ability of Aβ42(G37V) to modulate Aβ42 aggregation and reduce its toxicity. The outcome of this study introduces a novel agent by co-assembling Aβ42 with its G37V variant. Unlike conventional strategies focused solely on inhibiting Aβ42 aggregation, the Aβ42(G37V) variant promotes a shift toward faster aggregation and larger aggregated mass, which may sequester Aβ42 into less harmful forms. By exploring this alternative pathway, we aim to provide new insights into therapeutic strategies for mitigating Aβ42 toxicity in Alzheimer’s diseases.

## 2. Results

### 2.1. Aggregation Kinetics of Aβ42, Aβ42(G37V), and a Mixture of Aβ42/Aβ42(G37V)

**Figure 1:**
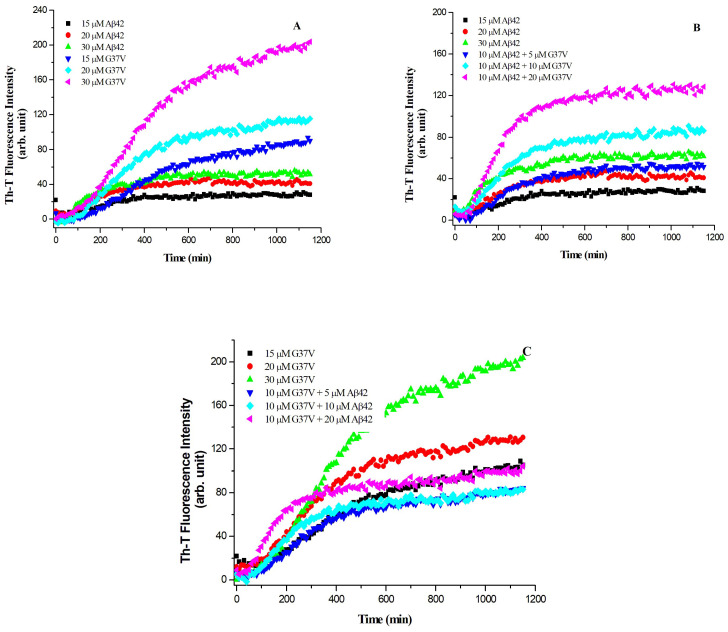
Aggregation kinetics of Aβ42, Aβ42(G37V), and their mixtures, as measured by Thioflavin-T (Th-T) fluorescence assay. (**A**) Aggregation of Aβ42(G37V) alone shows a significantly accelerated rate and higher Th-T fluorescence intensity compared to Aβ42 at equivalent concentrations, indicating faster aggregation. (**B**) When Aβ42(G37V) is added to a 10 μM Aβ42 solution, the mixture displays an increased aggregation rate compared to Aβ42 alone, suggesting that Aβ42(G37V) enhances the aggregation of Aβ42. (**C**) Introduction of varying concentrations of Aβ42 into a 10 μM Aβ42(G37V) solution results in slower aggregation kinetics compared to Aβ42(G37V) alone, indicating a concentration-dependent interaction between Aβ42 and Aβ42(G37V).

We utilized a Th-T fluorescence assay to investigate the aggregation rate by incubating Aβ42 with Aβ42(G37V) at various molar ratios and vice versa. Essentially, the aggregation process of Aβ42(G37V) exhibited a noteworthy acceleration and vigorous Th-T fluorescence intensity compared to that of Aβ42, both at equivalent concentrations (Figure 1A). When adding various concentrations of Aβ42(G37V) to a solution containing 10 μM of Aβ42, the resulting aggregation rates of the mixture were markedly faster than those of Aβ42 only (Figure 1B). In contrast, when various concentrations of Aβ42 were introduced to 10 μM of Aβ42(G37V) solution, the aggregation rates of the Aβ42(G37V)/Aβ42 mixture were considerably more gradual compared to those seen in Aβ42(G37V) alone at equivalent concentration (Figure 1C). These findings indicate that Aβ42(G37V) has the capability to expedite the aggregation rate of Aβ42.

### 2.2. Aggregation State Analyses

**Figure 2:**
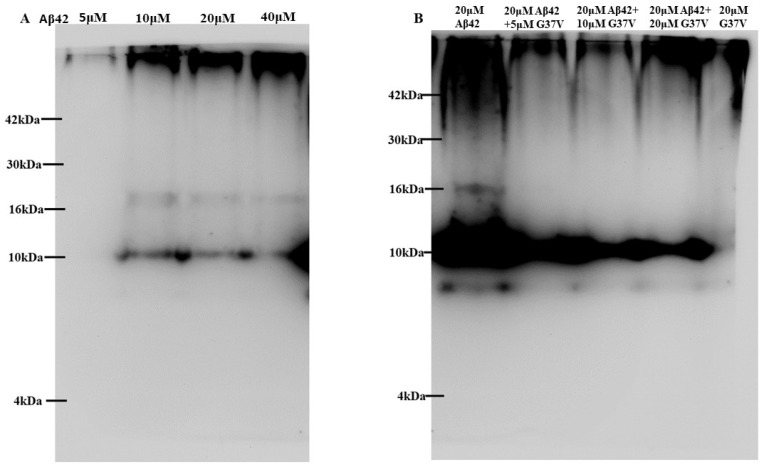
Western blot analysis of the aggregation profiles of Aβ42 and Aβ42(G37V) using a 6E10 monoclonal antibody. (**A**) Aβ42 alone shows a range of molecular masses, indicative of various aggregate sizes, with higher concentrations leading to a shift toward higher-molecular-weight aggregates. (**B**) When Aβ42(G37V) is co-incubated with Aβ42, the aggregation profiles shift toward higher molecular masses, with an increased presence of high-molecular-weight aggregates as the molar ratio of Aβ42(G37V) increases. This suggests that Aβ42(G37V) promotes the formation of larger Aβ42 aggregates.

As shown in the aggregation kinetics (Figure 1), adding Aβ42(G37) can accelerate the aggregation rate of Aβ42. To further investigate the aggregation state, we performed a Western blot analysis to assess the molecular weight distribution of aggregates formed by Aβ42, Aβ42(G37V), and their mixtures (Figure 2) using the Aβ1-16 monoclonal antibody (6E10).

Figure 2A shows the aggregation profile of Aβ42 alone at varying concentrations. The results show a broad distribution of molecular weights, with distinct bands corresponding to different sizes of Aβ42 aggregates. As the concentration of Aβ42 increases, a clear shift toward higher-molecular-weight species indicates the formation of larger aggregates. This confirms that Aβ42 aggregates into a wide range of molecular weight species, likely including toxic oligomers and larger fibrils.

In contrast, Figure 2B shows the aggregation profile of Aβ42 when co-incubated with Aβ42(G37V). Notably, the presence of Aβ42(G37V) leads to a significant shift in the molecular weight distribution of Aβ42 aggregates. Higher-molecular-weight aggregates become more prominent, and the lower molecular weight species associated with more toxic oligomers are less abundant. This indicates that Aβ42(G37V) promotes the rapid assembly of Aβ42 into larger, less toxic aggregates, thereby reducing the presence of smaller, potentially more harmful species.

These results suggest that Aβ42(G37V) alters the typical aggregation pattern of Aβ42, favoring the formation of high-molecular-weight aggregates that may sequester Aβ42 into less toxic forms.

### 2.3. Co-Assembly of Aβ42(G37V) and Aβ42

**Figure 3:**
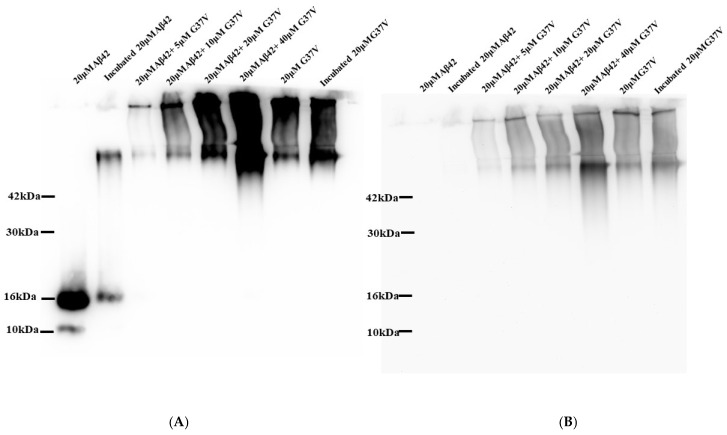
Analysis of the interaction between Aβ42 and Aβ42(G37V) using a biotin-conjugated pull-down assay. (**A**) Western blot analysis with a monoclonal antibody specific to Aβ42 shows that when Aβ42(G37V) is co-incubated with Aβ42, the latter shifts to higher-molecular-weight aggregates, consistent with the findings in Figure 2. Lanes 1 and 2 show Aβ42 alone, lanes 3–6 show Aβ42/Aβ42(G37V) mixtures, and lanes 7 and 8 show Aβ42(G37V) alone. (**B**) Western blot analysis using an antibody against biotin reveals that biotin-labeled Aβ42(G37V) coexists with Aβ42 in higher-molecular-weight aggregates, confirming their interaction and co-assembly.

To confirm whether the aggregation of Aβ42 in the presence of Aβ42(G37V) is due to direct interaction between the two peptides, we conducted a co-precipitation assay using biotin-labeled Aβ42(G37V) (Figure 3).

Figure 3A shows the results of Western blot analysis using a 6E10 monoclonal antibody. The lanes display Aβ42 alone (lanes 1 and 2), mixtures of Aβ42 and Aβ42(G37V) (lanes 3–6), and Aβ42(G37V) alone (lanes 7 and 8). When Aβ42(G37V) is added to Aβ42, we observe a clear shift in the aggregation profile toward higher molecular weights, consistent with the findings from Figure 2. This further supports the idea that Aβ42(G37V) accelerates the aggregation of Aβ42, leading to the formation of larger-molecular-weight aggregates.

Figure 3B, which uses a biotin-specific monoclonal antibody, confirms the interaction between Aβ42 and Aβ42(G37V). The protein bands show that biotin-labeled Aβ42(G37V) coexists with Aβ42 in the same high-molecular-weight aggregates. This finding demonstrates that Aβ42 and Aβ42(G37V) are co-assembled, and their interaction drives the formation of these larger aggregates.

Together, the results from Figure 2 and Figure 3 indicate that Aβ42(G37V) not only alters the aggregation kinetics of Aβ42 but also promotes the co-assembly of the two peptides into larger, less toxic aggregates. This co-assembly reduces the availability of smaller, toxic oligomers, suggesting a protective effect of Aβ42(G37V) against Aβ42-induced toxicity.

### 2.4. Morphological Characterization by AFM and TEM

**Figure 4:**
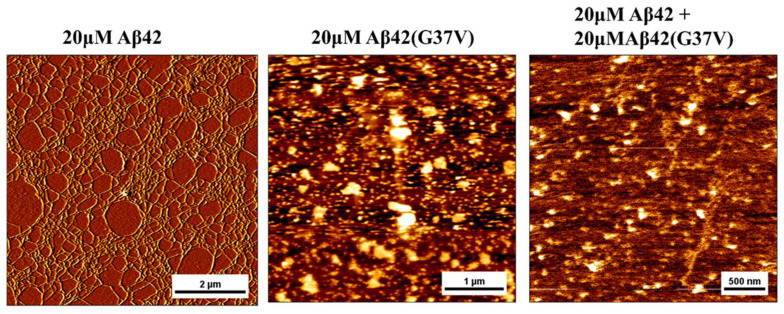
Atomic force microscopy (AFM) images show Aβ42 and Aβ42(G37V) morphological characteristics and their mixtures. Aβ42 alone forms network-like fibrillar structures typical of amyloid aggregates. In contrast, Aβ42(G37V) and the Aβ42/Aβ42(G37V) mixtures form aggregates with more rounded or elliptical shapes, suggesting that Aβ42(G37V) alters the aggregation morphology of Aβ42.

Since our previous study showed that Aβ42(G37V) aggregates form an elliptical shape instead of a typical network-like fibril structure [20], atomic force microscopy (AFM) was employed to investigate the morphologies of Aβ42, Aβ42(G37V), and the Aβ42/Aβ42(G37V) mixture, as depicted in Figure 4. The AFM images of Aβ42 alone displayed the characteristic network-like fibril structure. In contrast, the morphologies of Aβ42(G37V) and the combined Aβ42/Aβ42(G37V) aggregates exhibited a more rounded or elliptical shape, which is similar to a previous study [20]. This observation suggests that Aβ42(G37V) can influence Aβ42 aggregation morphologies, prompting it to adopt a distinct morphology.

**Figure 5:**
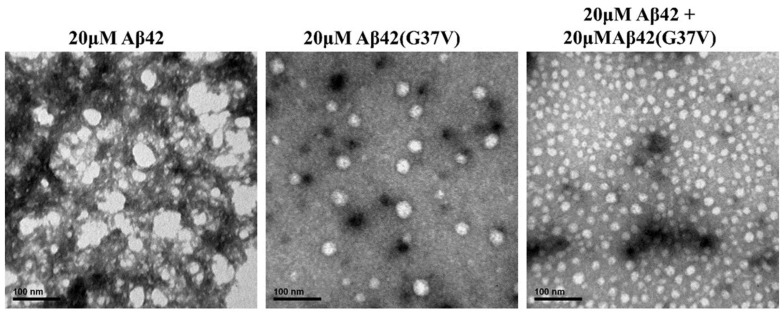
Transmission electron microscopy (TEM) images of Aβ42, Aβ42(G37V), and their mixtures. Aβ42 alone forms typical fibrillar structures, while Aβ42(G37V) and the Aβ42/Aβ42(G37V) mixtures predominantly form rounded aggregates. These morphological differences suggest that Aβ42(G37V) induces a shift from the fibrillar morphology of Aβ42 to a less ordered, potentially less toxic structure.

Additionally, the morphologies of Aβ42, Aβ42(G37V), and Aβ42 combined with Aβ42(G37V) were assessed using transmission electron microscopy (TEM), as showcased in Figure 5. Consistent with the AFM findings (Figure 4), the morphology of Aβ42 aggregates presented a network-like fibril pattern. On the other hand, both Aβ42(G37V) and the mixture of Aβ42 with Aβ42(G37V) exhibited a rounded shape. This alignment with the AFM observations reinforces the concept that Aβ42(G37V) instigates a morphological shift in Aβ42 during the progression of aggregation. The results underscore the notion that Aβ42(G37V) has the capability to prompt a transformation in the morphological arrangement of Aβ42 throughout aggregation.

### 2.5. Analyses of Secondary Structure by ATR-FTIR

**Figure 6:**
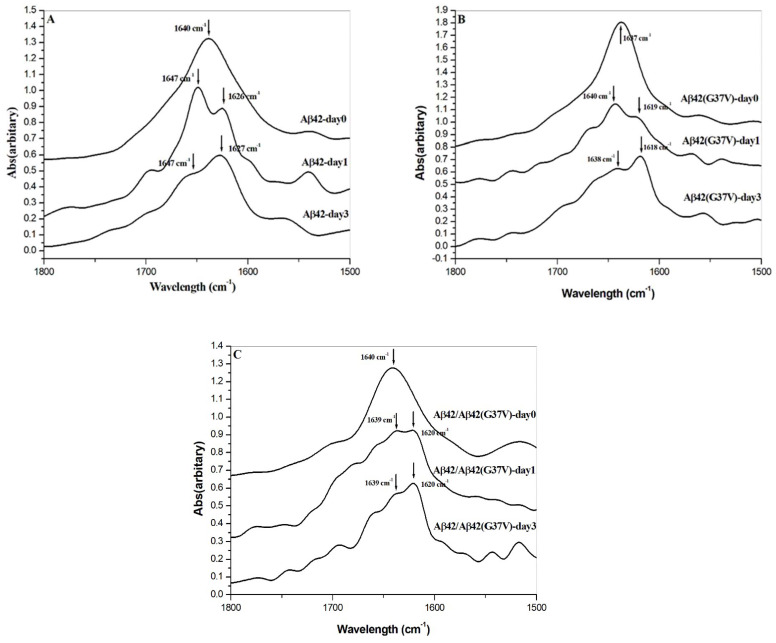
ATR-FTIR analysis of the secondary structure evolution in Aβ42, Aβ42(G37V), and their mixtures over time. (**A**) On day 0, the spectra display a broad peak at ~1640 cm^−1^, indicating a predominantly disordered structure. (**B**) By day 1, a shift toward a prominent β-sheet structure is observed for Aβ42, with a peak emerging at 1626 cm^−1^, further intensifying by day 3. (**C**) A similar shift occurs for Aβ42(G37V) and its mixture with Aβ42, but with a slight downshift to 1620 cm^−1^, indicating altered β-sheet packing. The spectral shifts observed in all samples reflect a transition to extended β-sheet conformations over time, with differences in packing and organization driven by the G37V substitution. This structural alteration supports the hypothesis that Aβ42(G37V) accelerates aggregation and forms larger, less toxic aggregates.

The ATR-FTIR spectroscopy-based analyses of the secondary structure for Aβ42, Aβ42(G37V), and the Aβ42/Aβ42(G37V) mixture at day 0, day 1, and day 3 are presented in Figure 6A–C, respectively. In the IR spectrum, the amide I peak in the range of 1630–1610 cm^−1^ and 1645–1630 cm^−1^ were assigned to represent intermolecular or extended β-sheet and intramolecular β-sheet conformations of Aβ, respectively [25,26].

On day 0, all IR spectra of Aβ42, Aβ(G37V), and Aβ42/Aβ42(G37V) peptides showed the absence of IR peaks below 1630 cm^−1^, with a broad band observed around 1640 cm^−1^ in the amide I region for all peptides. This observation suggests these peptides initially adopt a β-sheet structure [22,23]. Moving to day 1, an IR peak at 1626 cm^−1^ emerged for Aβ42 alone, becoming more pronounced by day 3 (Figure 6A). For Aβ(G37V) and Aβ42/Aβ42(G37V) peptides, the major IR peaks shifted from 1640 cm^−1^ on day 0 to 1620–1618 cm^−1^ on day 1 and day 3 (Figure 6B,C). Similar to Aβ42, the 1620 or 1618 cm^−1^ peaks are more profound on day 3 for both Aβ(G37V) and Aβ42/Aβ42(G37V) peptides. These findings indicate that the Aβ42, Aβ(G37V), and Aβ42/Aβ42(G37V) mixtures can all form an extended β-sheet structure over time.

### 2.6. Cytotoxicity Analysis

**Figure 7:**
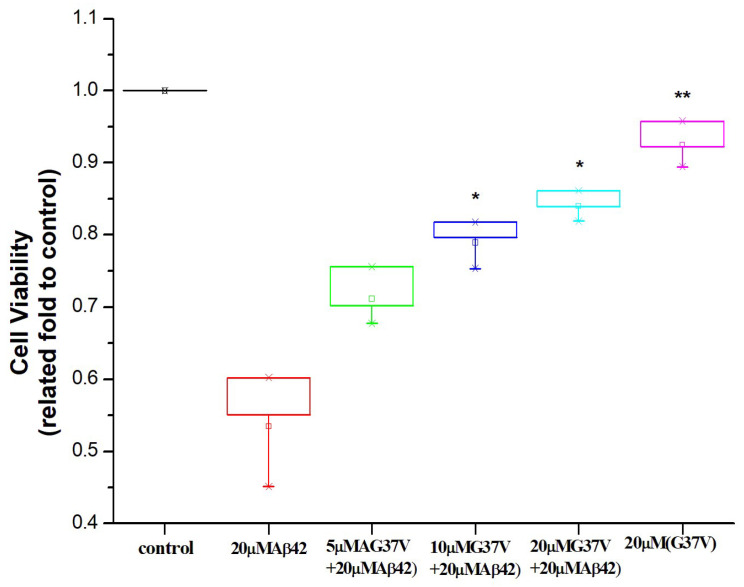
Analysis of cell viability following treatment with Aβ42, Aβ42(G37V), and their mixtures. Treatment with 20 μM Aβ42 alone reduces cell viability (<60%) compared to control cells. In contrast, 20 μM Aβ42(G37V) maintains cell viability above 80%. Co-incubation of Aβ42 with increasing concentrations of Aβ42(G37V) enhances cell viability, with survival rates exceeding 80% at higher Aβ42(G37V) concentrations. These results indicate that Aβ42(G37V) mitigates the cytotoxic effects of Aβ42 (*n* = 3, mean ± std, * *p* ≤ 0.05, ** *p* ≤ 0.001, related to 20 μM Aβ42).

As unveiled within this study, introducing Aβ42(G37V) to Aβ42 leads to aggregation rate, configuration, and morphology alterations. Subsequently, we evaluated the impact of varying concentrations of Aβ42(G37V) on the cytotoxicity of Aβ42. Figure 7 compares cell survival rates after treatment with 20 μM of Aβ42 and Aβ42(G37V) alone and with mixing diverse Aβ42(G37V) concentrations with 20 μM Aβ42 over a 72 h interval.

Upon administering 20 μM of Aβ42 alone, the cell viability was less than 60% compared to the control group (without treating any Aβ peptides). In contrast, the cell survival rate surpassed 80% when administering 20 μM of Aβ42(G37V) alone. Importantly, including Aβ42(G37V) alongside Aβ42 demonstrated a noticeable enhancement in cell viability that correlated with the increasing concentrations of Aβ42(G37V). Notably, the cell viabilities within the range of introducing 10 to 20 μM of Aβ42(G37V) into 20 μM Aβ42 solution were consistently above 80%. These findings substantiate that the Aβ42 aggregation states and morphological changes by Aβ42(G37V) effectively counteract the cytotoxic consequences associated with Aβ42.

### 2.7. Reactive Oxygen Species (ROS) and Lipid Peroxidation Analysis

**Figure 8:**
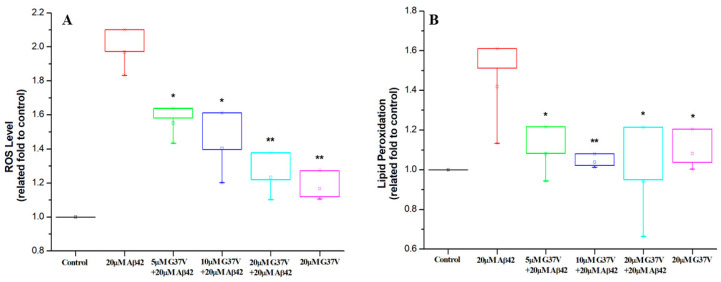
Analysis of reactive oxygen species (ROS) production and lipid peroxidation in cells treated with Aβ42, Aβ42(G37V), and their mixtures. (**A**) Treatment with 20 μM Aβ42 alone significantly increases ROS production, while co-incubation with increasing concentrations of Aβ42(G37V) dose-dependently reduces ROS levels. (**B**) Similarly, lipid peroxidation, which is elevated by 20 μM Aβ42, is reduced when Aβ42(G37V) is added to the mixture, indicating that Aβ42(G37V) mitigates oxidative stress and lipid peroxidation induced by Aβ42 (*n* = 3, mean ± std, * *p* ≤ 0.05, ** *p* ≤ 0.001, related to 20 μM Aβ42).

One of the mechanisms implicated in the cytotoxicity of Aβ pertains to the generation of reactive oxygen species (ROS). Subsequently, we explored whether the reduction in Aβ42 cytotoxicity achieved by introducing Aβ42(G37V) is linked to a decline in ROS levels. Upon introducing concentrations of 5, 10, and 20 µM of Aβ42(G37V), a noticeable downward trend in the proportion of ROS emerged, corresponding with the increasing concentration of Aβ42(G37V) (as shown in Figure 8A).

For 20 μM Aβ42 alone, the proportion of ROS was nearly twice as high as that of the control group (without treating any Aβ peptides). When treated with 5, 10, and 20 µM of Aβ42(G37V) in a 20 μM Aβ42 solution, the levels of ROS were 1.6-, 1.4-, and 1.25-fold higher, respectively, than the control group. Our findings demonstrate that adding Aβ42(G37V) significantly reduces the formation of ROS induced by Aβ42.

In addition to inducing ROS production, Aβ42 can cause lipid peroxidation. Therefore, we further investigated the effect of adding Aβ42(G37V) to Aβ42 on the production of lipid peroxidation. Figure 8B illustrates the protective effect of Aβ42(G37V) against lipid peroxidation induced by Aβ42. Similar to the ROS levels, the introduction of Aβ42(G37V) was found to reduce lipid peroxidation caused by Aβ42. The level of lipid peroxidation was 1.42-, 1.10-, 1.04-, and 0.94-fold higher than that of the control group, respectively, indicating that the level of lipid peroxidation induced by Aβ42 is significantly reduced by the addition of Aβ42(G37V).

Taken together, the outcomes demonstrate that introducing Aβ42(G37V) to Aβ42 can effectively provide cellular protection against the toxic effects of Aβ42 by suppressing ROS production and lipid peroxidation.

## 3. Discussion

Alzheimer’s disease (AD) is a complex neurodegenerative disorder characterized by progressive cognitive impairments and the accumulation of pathological hallmarks, including amyloid-beta (Aβ) plaques and tau neurofibrillary tangles [2,3]. The amyloid cascade hypothesis has long been a central theory in AD research, positioning Aβ peptides at the core of disease pathogenesis [2]. These peptides can adopt a β-strand conformation, facilitating their aggregation into oligomers, fibrils, and plaques—structures capable of inducing neuronal dysfunction and apoptosis [5,6,7,8,9,10]. As such, strategies that prevent the formation of toxic Aβ aggregates are highly sought after for AD prevention and treatment.

Traditional approaches to preventing Aβ42 toxicity have primarily focused on inhibiting Aβ42 aggregation using small molecules, such as vitamin K3 [13] and curcumin [12], which have demonstrated anti-amyloidogenic effects. Various anti-amyloidogenic peptides have also been designed to block Aβ aggregation [27,28,29,30,31,32,33]. These strategies typically focus on interfering with the self-recognition domains of Aβ [27,28], utilizing random peptide sequences [29,30,31] or mimicking Aβ-binding proteins to inhibit fibrillogenesis [32,33]. The most common targets for such interventions include vital regions like the K16LVFF20 core domain or discordant helix region, which play essential roles in Aβ aggregation [27,28]. Despite these promising findings, the conventional approach of inhibiting Aβ42 aggregation may not always result in an effective therapeutic response, particularly when forming larger, less toxic aggregates is a more viable solution. In contrast to this inhibitory approach, we propose an alternative strategy to mitigate Aβ42 toxicity by modifying its aggregation profile by introducing a mutant variant, Aβ42(G37V), to shift the balance away from small toxic aggregates toward less harmful large aggregates.

As shown in the current study, the biotin pull-down assay confirmed that Aβ42(G37V) can interact with Aβ42. Western blot analysis revealed that introducing Aβ42(G37V) can predominantly shift the Aβ42 to higher-molecular-weight aggregates. Thioflavin-T (Th-T) assays further show that Aβ42(G37V) accelerates the aggregation rate of Aβ42. Putting all these results together, our present studies suggest that introducing Aβ42(G37V) into Aβ42 solutions accelerates aggregation kinetics and favors the rapid formation of larger, higher-molecular-weight aggregates, suggesting that Aβ42(G37V) can drive Aβ42 toward forming higher-molecular-weight species and likely limits the time during which smaller aggregates can form and persist. These findings align with previous studies [14,34], such as those by Hung et al., which showed that substitutions in the glycine-zipper motif, mainly replacing glycine with leucine, can expedite the aggregation process [14].

Soluble small aggregates are known to interact with neuronal membranes, induce calcium dysregulation, and trigger oxidative stress by generating reactive oxygen species (ROS) [9,10,15,16,17]. By promoting the rapid assembly of larger aggregates, Aβ42(G37V) minimizes the presence of these toxic intermediates, thereby reducing their neurotoxic potential. This mechanistic insight highlights the profound impact of Aβ42(G37V) on the aggregation properties of Aβ42.

Our study further demonstrates that Aβ42(G37V) alters not only the aggregation mass and rate but also the morphology of the aggregates. AFM and TEM images revealed a shift from the typical fibrillar structures of Aβ42 to more rounded or elliptical forms characteristic of Aβ42(G37V) by introducing Aβ42(G37V). The ATR-FTIR analyses confirmed that, despite the observed morphological differences, all Aβ peptides, whether wild-type Aβ42, Aβ42(G37V), or their mixtures, adopt an extended β-sheet conformation. This finding suggests that the G37V substitution does not prevent the formation of β-sheets, which are crucial for amyloid aggregation but alters the structural organization and packing of these β-sheets.

The possible cause for the changes in Aβ42 morphology by introducing Aβ42(G37V) is the aggregation nature of Aβ42(G37V). Our previous study demonstrated that substituting glycine with the bulkier, hydrophobic valine at position 37 introduces steric hindrance, increases hydrophobicity, and destabilizes the electrostatic interaction between the Asp23 and Lys 28 salt bridge [24]. Positioned within the glycine-zipper motif, a critical structural element consisting of glycine residues at positions G25, G29, G33, and G37, it typically promotes close packing of β-strands within Aβ fibrils due to the small size and flexibility of glycine, enabling tight interactions between β-sheets. By interacting with Aβ42(G37V), the packing of Aβ42 β-strands becomes less compacted and forms large and spherical aggregates compared to the fibrillar structures observed with wild-type Aβ42, as revealed by the AFM and TEM analyses.

The increased flexibility or destabilization of this salt bridge caused by introducing Aβ42(G37V) is likely to promote the rapid aggregation of Aβ42 toward forming larger, less toxic aggregates, as reflected by Western blot assays. These structural changes likely reduce the formation of smaller, more toxic oligomers, thereby diminishing the interaction of Aβ42 with neuronal membranes and ultimately reducing cytotoxicity since the larger aggregates tend to interact with neuronal membranes less than the smaller aggregates do [17,35]. This shift away from smaller toxic aggregates to larger and globular aggregates may explain the reduced cytotoxicity and be likely further responsible for reducing the production of reactive oxygen species (ROS) and lipid peroxidation observed in this study, as the smaller and more toxic species are the main drivers of oxidative stress.

Oxidative stress plays a critical role in the neurodegenerative processes underlying Alzheimer’s disease (AD). Accumulating evidence suggests that reactive oxygen species (ROS) are major contributors to Aβ42-induced toxicity, as elevated ROS levels can lead to mitochondrial dysfunction, lipid peroxidation, protein oxidation, and DNA damage, all of which contribute to neuronal death [9,10,11,15,16,17]. The link between Aβ aggregation and oxidative stress is well-established, with smaller, soluble Aβ aggregates known to induce more significant levels of oxidative damage than larger aggregates [35].

In this study, we observed that introducing the Aβ42(G37V) variant significantly reduces ROS production induced by Aβ42, as demonstrated by biochemical assays. The mechanism by which Aβ42(G37V) exerts this protective effect is likely linked to its ability to alter the aggregation pathway of Aβ42, promoting the formation of larger, less toxic aggregates. By accelerating the aggregation of Aβ42 and limiting the presence of smaller toxic species, Aβ42(G37V) reduces the capacity of Aβ42 to interact with neuronal membranes and induce oxidative damage.

The reduction in lipid peroxidation, a process driven by ROS-induced damage to cellular membranes, further supports the protective role of Aβ42(G37V). Lipid peroxidation is known to disrupt membrane integrity, alter ion homeostasis, and trigger apoptotic pathways, all contributing to neuronal dysfunction in AD. By reducing lipid peroxidation, Aβ42(G37V) helps preserve membrane integrity and prevent the cascade of events leading to cell death.

The decrease in ROS production and lipid peroxidation is particularly significant, as oxidative stress is a major contributor to neuronal damage in AD [9,10,15,17]. By promoting the formation of larger, less toxic aggregates, introducing Aβ42(G37V) reduces the capacity of Aβ42 to induce oxidative stress, suggesting a protective role for this variant. This reduction in oxidative damage further supports the idea that modulating Aβ42 aggregation, rather than simply inhibiting it, could be a viable strategy to mitigate its neurotoxic effects.

The significance of these findings lies in the broader role of oxidative stress in AD pathology. Elevated ROS levels and lipid peroxidation have been implicated in the progression of AD, with oxidative damage contributing to synaptic loss, mitochondrial dysfunction, and neuronal death. By modulating Aβ42 aggregation and reducing oxidative damage, Aβ42(G37V) offers a potential therapeutic strategy that targets both the aggregative and oxidative aspects of Aβ42 toxicity. This dual mechanism of action underscores the importance of addressing oxidative stress in AD treatment alongside a strategy aimed at reducing Aβ aggregation.

The results of this study provide new insights into the potential for Aβ42(G37V) to modulate Aβ42 aggregation as a therapeutic strategy for Alzheimer’s disease. Unlike traditional approaches that focus on inhibiting Aβ42 aggregation altogether, the G37V variant accelerates the aggregation process but directs it towards the formation of larger, less toxic aggregates. By reducing the presence of smaller, soluble oligomers, which are known to induce membrane disruption, oxidative stress, and neuronal death, Aβ42(G37V) may offer a novel therapeutic pathway.

Given that oxidative stress and lipid peroxidation are key drivers of neuronal damage in Alzheimer’s disease, the ability of Aβ42(G37V) to reduce ROS production and lipid peroxidation makes it a strong candidate for therapeutic development. Moreover, this variant may be combined with antioxidants or anti-tau therapies to enhance neuroprotection. These results open the door for future studies to explore the synergistic effects of Aβ42(G37V) with other treatment modalities, potentially improving outcomes for patients with Alzheimer’s disease.

Additionally, in vivo studies will provide insights into the long-term effects of promoting larger, less toxic aggregates and whether this agent prevents synaptic loss and neurodegeneration. The potential for Aβ42(G37V) to reduce tau pathology and neuroinflammatory responses must also be explored, as Aβ interacts with other pathological mechanisms in Alzheimer’s disease. Future work should also systematically examine other variants targeted on the glycine-zipper motif for their potential and insight to reduce the Aβ42 cytotoxicity.

In conclusion, this study demonstrates that the Aβ42(G37V) variant offers a promising new agent to mitigate Aβ42 toxicity by promoting the rapid formation of larger, less harmful aggregates. By shifting the aggregation pathway away from toxic oligomers, introducing Aβ42(G37V) reduces oxidative stress, lipid peroxidation, and overall cytotoxicity induced by toxic Aβ42 aggregates. These findings suggest that modulating, rather than inhibiting, Aβ aggregation could serve as a viable therapeutic strategy for Alzheimer’s disease. Further studies are warranted to explore the full potential of this agent in in vivo models and to investigate its application in combination with other therapies targeting multiple pathways in AD progression.

## 4. Materials and Methods

### 4.1. Materials

The wild-type Aβ42, Aβ42(G37V), and biotin-labeled Aβ42(G37V) peptides were synthesized by Yao-Hong Biotechnology Inc. (Taiwan) using solid-phase synthesis. Subsequently, the peptides were purified using high-performance liquid chromatography (HPLC) and confirmed to have a purity of ≥95% through mass spectrometry analysis. The peptides were directly utilized in all experimental procedures without further modifications after purification.

### 4.2. Aggregation Kinetics

Aggregation kinetics of the wild-type Aβ42, Aβ42(G37V), and their mixtures were assessed using the Thioflavin-T (Th-T) fluorescence assay. Stock solutions of Aβ peptides were prepared by dissolving 1 mg of peptide in 0.5 mL of 0.1N NaOH and were stored at −150 °C until use. The stock solutions were diluted to the desired concentration (15, 20, and 30 μM for Aβ42 and Aβ42(G37V) each alone, 10 μM Aβ42/5, 10, and 20 μM Aβ42(G37V), and 10 μM Aβ42(G37V)/5, 10, and 20 μM Aβ42 mixtures) in 25 mM phosphate buffer (pH 7.4) for aggregation assays, supplemented with 5 μM Thioflavin-T and 0.01% NaN3. The Th-T fluorescence, indicative of β-sheet formation during peptide aggregation, was measured at 10 min intervals using a microplate reader (FlexStation 3, Molecular Devices, San Jose, CA, USA) at 37 °C, with an excitation wavelength of 450 nm and emission at 490 nm. Aggregation kinetics were plotted as the average of three independent replicates.

### 4.3. Aggregation State Analysis

Aggregation state analyses were performed on Aβ42, Aβ42(G37V), and their mixtures at specified concentrations and molar ratios (5, 10, 20, and 40 μM Aβ42, and 20 μM Aβ42/5, 10, and 20 μM Aβ42(G37V) mixtures). Peptides were dissolved in phosphate buffer (pH 7.0) and incubated for 24 h at 37 °C. The samples were then subjected to 10% native Tricine-PAGE and transferred onto polyvinylidene difluoride (PVDF) membranes (0.22 µm, PE) over 2 h. Membranes were blocked with 5% nonfat milk in phosphate-buffered saline (PBS) for 1 h and subsequently incubated overnight at 4 °C with a primary anti-mouse monoclonal antibody against Aβ1-16 (6E10, Abbiotec, LLC., San Diego, CA, USA; 1:2000 dilution). Following primary antibody incubation, membranes were washed three times with PBST and incubated with a goat anti-mouse secondary antibody (Sigma, Poole, UK; 1:6000 dilution). Chemiluminescent detection was conducted using a chemiluminescent kit (GE, Pittsburgh, PA, USA), and imaging was performed using a CCD camera system (UVP, Rockland Immunochemical Inc., Limerick, PA, USA). Blot images were analyzed using the ImageJ software (version 1.53t).

### 4.4. Cross-Interaction Analysis Using Co-Precipitation

To investigate the interaction between Aβ42(G37V) and Aβ42, co-precipitation experiments were performed using a BcMag™ Streptavidin Magnetic Beads kit (Bioclone Inc., San Diego, CA, USA). A 20 μM mixture of Aβ42 and biotin-labeled Aβ42(G37V) at predetermined molar ratios (5, 10, and 20 μM Aβ42(G37V)) was incubated at 37 °C for 24 h. Fifty microliters of the incubated samples containing either Aβ42, Aβ42(G37V), or Aβ42/biotin-labeled Aβ42(G37V) were mixed with streptavidin-coated magnetic beads in 1.0 mL of binding buffer (PBS, 0.1% BSA, pH 7.4) and incubated for 30 min at room temperature with rotational mixing. The samples were then placed in a magnetic separator for 3 min, allowing supernatants to be removed and discarded. The pellet was resuspended in 1.0 mL of fresh binding buffer, and this wash process was repeated three times. The final pellet was dissolved in 0.1 mL of binding buffer, separated by 10% SDS-PAGE, and transferred onto a PVDF membrane (0.22 µm, PE) for subsequent Western blot analysis.

The blotting procedures were similar to the aggregation state analyses in Section 4.3, except that the Aβ42 primary anti-mouse monoclonal antibody (Abcam, Cambridge, UK; 1:2000 dilution) and biotin primary anti-mouse monoclonal antibody (Abcam, Cambridge, UK; 1:5000 dilution) were used to analyze Aβ42 and biotin-labeled Aβ42(G37V), respectively. To avoid any artificial or false results, the Western blots were first detected using the Aβ42 antibodies. Then, after bleaching the Aβ42 antibodies, the same blots were detected using the biotin antibody. The Western blots were detected using a goat anti-mouse secondary antibody (Sigma, Poole, UK; 1:6000 dilution) and a chemiluminescent kit (GE, Pittsburgh, PA, USA). The image was detected using a CCD camera system (UVP, Rockland Immunochemical Inc., Limerick, PA, USA) and analyzed using the ImageJ program.

### 4.5. Morphological Analyses

Aggregation morphologies of Aβ42 (20 μM), Aβ42(G37V) (20 μM), and their mixtures (20 μM/20 μM) were characterized using transmission electron microscopy (TEM) and atomic force microscopy (AFM). Peptide samples were incubated for 24 h before analysis. Ten microliters of each sample were deposited onto a cleaved mica disc (Ted Pella Inc., Redding, CA, USA) for AFM imaging or onto a carbon-coated 200-mesh copper grid (Ted Pella Inc., Redding, CA, USA) for TEM analysis.

AFM images were acquired in contact mode using a Nanowizard™ AFM instrument (JPK Instruments, Berlin, Germany) installed on an inverted optical microscope (Nikon Corporation, Tokyo, Japan). The AFM probes used were oxide-sharpened silicon nitride probes (OMCL-TR400PB-1, Olympus, Tokyo, Japan) with a spring constant of 0.02 N/m. Images were captured at a 1–2 Hz scanning rate with a resolution of 512 × 512 pixels. Image processing and analysis were performed using SPM software v. 3.16 (Nanowizard™).

TEM images were analyzed using transmission electron microscopy (Hitachi model H-7650, Tokyo, Japan) with an accelerating voltage of 100 keV. The grids containing samples were stained with 2 μL of 2% uranyl acetate for 30 s and air-dried for 30 min before TEM measurement.

### 4.6. Analysis of Fourier-Transform Infrared Spectroscopy

To analyze the secondary structure of Aβ42, Aβ42(G37V), and the Aβ42/Aβ42(G37V) mixture both before and after incubation, we utilized a Fourier-transform infrared (FTIR) spectrometer (Jasco, FTIR/4100, Tokyo, Japan) equipped with an attenuated total reflection (ATR) accessory. This instrument facilitated the examination of conformational changes in Aβ peptides during the aggregation process.

In the sample preparation process, we incubated 30 μL of Aβ42 (20 μM), Aβ42(G37V) (20 μM), and the Aβ42/Aβ42(G37V) mixture (20 μM/20 μM) at 37 °C for 0 (day 0), 24 (day 1), and 72 h (day 3). Subsequently, these samples were applied onto a ZnSe crystal and allowed to desiccate overnight in desiccators at room temperature. The spectra were recorded in the 1800–1400 cm^−1^ wavelength range with a 2 cm^−1^ interval. Three replicates were recorded, and the data were later smoothed using a Savitsky–Golay function in Origin 6.0 software.

Peak identification was carried out by analyzing the first derivative of the IR spectrum within the amide I region. The secondary structure analysis was conducted using the deconvolution function in Origin 6.0 software.

### 4.7. Cell Viability Assay

The synthesized Aβ peptides were prepared as a 500 μM stock solution in 0.1N NaOH. These peptide stock solutions were then diluted to the designed concentrations in 25 mM phosphate buffer (pH 6.8) and incubated at 4 °C overnight for the cell survival assay. For cell culture, 5 × 10^5^ of C6 cells in each well of a 96-well microtiter plate were cultured in a culture medium with the Aβ42 (20 μM), Aβ42(G37V) (20 μM), and the Aβ42/Aβ42(G37V) mixture (20 μM Aβ42/5, 10, and 20 μM Aβ42(G37V)) at the designed concentrations for 72 h at 37 °C. The same culture condition without Aβ peptides was used as a control. Ten μL of MTT solution was added to each well for the cell survival assay and further incubated for another 4 h at 37 °C. The absorbance at a wavelength of 570 nm was used to measure the cell survival rate.

### 4.8. Reactive Oxygen Species (ROS) Assay

The fluorescent reporter dye 30-(p-hydroxyphenyl) fluorescein (5 mM) was used to detect hydroxyl radical formation. A total of 5 × 10^5^ C6 cells were cultured in a culture medium without or with the Aβ42, Aβ42(G37V), and the Aβ42/Aβ42(G37V) mixture at the designed concentrations for 24 h at 37 °C. The C6 cells were lysed and mixed with 10 μL of fluorescein. The fluorescence intensity at an emission wavelength of 545 nm with an excitation wavelength of 488 nm was used to determine the related fold of hydroxyl radical using a microplate reader (FlexStation 3, Molecular Device Inc., San Jose, CA, USA).

### 4.9. Lipid Peroxidation Assay

The lipid peroxidation was measured using the PEROXsayTM-Lipid kit (G-Biosciences, St. Louis, MO, USA). A total of 5 × 10^5^ C6 cells were cultured in a culture medium without or with the Aβ42 (20 μM), Aβ42(G37V) (20 μM), and the Aβ42/Aβ42(G37V) mixture (20 μM Aβ42/5, 10, 20 μM Aβ42(G37V)) at the designed concentrations for 24 h at 37 °C. The C6 cells were lysed and resuspended with 200 μL of kit assay solution, prepared by mixing 1 volume of component 2 with 100 volumes of component 1 in a 96-well microplate. The solution was further incubated at room temperature for 30 min. The absorbance at a wavelength of 595 nm was used to determine the related fold of lipid peroxidation using a microplate reader (FlexStation 3, Molecular Device Inc., San Jose, CA, USA).

### 4.10. Statistical Analysis

All experiments were performed in triplicate (*n* = 3), and the data are presented as mean ± standard deviation (SD). Statistical analysis was conducted using one-way analysis of variance (ANOVA) followed by Tukey’s post hoc test to evaluate the significance of differences between experimental groups. For datasets involving multiple comparisons (e.g., ROS production, lipid peroxidation, and cell viability assays), a *p*-value of ≤0.05 was considered statistically significant. All statistical analyses were performed using Original 6.0 software.

## Data Availability

The data are made available at the request of the corresponding author.
